# Function of Glial Cells in Neuroinflammatory and Neuroimmunological Responses II

**DOI:** 10.3390/cells12131750

**Published:** 2023-06-30

**Authors:** Ruqayya Afridi, Anup Bhusal, Makoto Tsuda, Hoon Ryu, Kyoungho Suk

**Affiliations:** 1 Department of Pharmacology, School of Medicine, Kyungpook National University, Daegu 41944, Republic of Korea; 2 Brain Korea 21 Four KNU Convergence Educational Program of Biomedical Sciences for Creative Future Talents, Kyungpook National University, Daegu 41940, Republic of Korea; 3 Department of Molecular and System Pharmacology, Graduate School of Pharmaceutical Sciences, Kyushu University, Fukuoka 812-8582, Japan; 4 Center for Neuroscience, Brain Science Institute, Korea Institute of Science and Technology (KIST), Seoul 02792, Republic of Korea; 5 Brain Science and Engineering Institute, Kyungpook National University, Daegu 41944, Republic of Korea

It is now well established that glial cells play an equal, if not greater, role in regulating intricate functions of the central nervous system (CNS) compared with neurons [1]. Glial cells are well known for their supportive role during neural cell development, and they also create an optimal environment for neurons to operate effectively, ensuring the appropriate conduction of impulses by axonal insulation, neurotransmitter release, and uptake, as well as metabolic support [1]. Conversely, in various pathological conditions within the nervous system, glial cells exhibit disruptions in their activity [2]. Although extensive research has shed light on several facets of glial cell behavior in a diverse range of neurological disorders, their involvement in regulating neuroinflammatory responses and their contribution to neuroimmune interactions have not been explored in detail. This Special Issue (SI) endeavors to present a comprehensive overview of the remarkable strides made in the realm of glia-centric research on neuroinflammation and neuroimmunology. The SI presents an interesting collection of four groundbreaking research articles and three comprehensive review articles, discussing the neuroinflammatory and neuroimmunological aspects of glia across diverse disease contexts.

Lead exposure is considered one of the causes of neurological disabilities in both children and adults [3]. The study by Liana Shvachiy et al. aimed to understand the molecular changes involved in the temporal remodeling of physiological functions during intermittent lead exposure [4]. They demonstrated that lead exposure in animals caused the inflammatory activation of microglia and astrocytes in the hippocampus with concomitant local neuroinflammation, consistent with changes in the reflex regulation of cardiovascular and respiratory parameters. They also observed that lead exposure resulted in a significant long-term episodic memory impairment in animals. Overall, these findings suggest that individuals who have been exposed to lead and subsequently develop chronic neuroinflammation present an increased susceptibility to adverse events, especially if they have a preexisting cardiovascular disease or are elderly.

Natural products have been known and used since ancient times for their therapeutic properties. Recent studies have demonstrated their potential as neuroprotective agents for the treatment of neurodegenerative diseases [5]. For instance, a study by Jang et al. showed that micrandilactone C, a nortriterpenoid isolated from the roots of *Schisandra chinensis*, could ameliorate 3-nitropropionic acid (3-NPA)-induced Huntington’s disease (HD)-like symptoms by inhibiting the STAT3 pathway [6]. The pretreatment of mice with micrandilactone C ameliorated the neurobehavioral disorder, improved the survival rate, and inhibited the neurodegeneration related to apoptosis in the striatum after 3-NPA intoxication. This evidence suggests that compounds from natural resources are beneficial for the treatment of neuroinflammatory conditions such as HD. Nevertheless, such studies are limited to animal models, and further research is necessary to confirm these findings. Kinases are key signaling components that regulate several cellular processes, including inflammation. Targeting kinases, such as receptor-interacting serine/threonine-protein kinase 1 (RIPK1), with small-molecule inhibitors is a promising strategy for treating neuroinflammatory diseases [7]. Kim et al. showed that RIPK1 inhibition using a specific small-molecule inhibitor suppressed microglial activation and proinflammatory marker expression in the brains of lipopolysaccharide (LPS)-injected mice [8]. Consistent with this, RIPK1 inhibition protected against dopaminergic neuronal cell death and inhibited microglial activation in a mouse model of Parkinson’s disease. The study also showed that RIPK1 inhibition exerted anti-inflammatory effects primarily by modulating the AMPK, PI3K/Akt, MAPKs, and NF-κB pathways in LPS-stimulated BV-2 mouse microglia cells. Although their findings are impressive, multiple kinases were involved in the same pathway and the kinase inhibitors can exert off-target effects. Furthermore, there are several other challenges, such as drug resistance, toxicity, and cost, that must be addressed before kinase inhibitors can be used to effectively treat a wider range of neuroinflammatory diseases.

Cathelicidins are key antimicrobial effector proteins that protect body surfaces from invasive bacterial infections as a component of the innate immune system [9]. Bhusal et al. demonstrated that the expression of cathelicidin-related antimicrobial peptide (CRAMP) was induced in multiple cell types, such as astrocytes, microglia, and neurons, during LPS-induced neuroinflammation [10]. Treatment with CRAMP reduced the proinflammatory effects of LPS in cultured glial cells and mice. They also showed that the ability of CRAMP to suppress LPS response in glia was not dependent on the sequence of exposure to CRAMP or LPS. These data suggest that CRAMP expression in the brain is a component of the innate immune response to bacterial infection, and CRAMP may act to prevent excessive neuroinflammation.

This SI also includes exciting reviews on microglial roles and their heterogeneity in stress-related depression and Alzheimer’s disease (AD) pathology, respectively. The review by Afridi et al. elegantly discusses the potential triggers of microglial inflammatory activation and its consequent roles in stress-induced depressive-like behaviors [11]. Microglial cells are emerging as crucial regulators of psychiatric disorders, particularly major depressive disorders [12]. The review presents a comprehensive analysis of current research on the potential relationship between microglia-mediated neuroinflammation and stress-induced depression. The authors provide valuable insights into the role of microglia in depression and stress-based animal models, emphasizing their contribution to inflammatory processes and maladaptive changes. The review also discusses the potential therapeutic targets for depression treatment based on modulating the activation and function of microglia.

In addition, the insightful review by Fujikawa et al. sheds light on the phenotypic and functional heterogeneity of microglia in AD pathology [13]. Considering the complex nature of microglial functions encompassing both detrimental and beneficial aspects, unraveling their precise roles has proven challenging. This review serves as a sophisticated endeavor to increase our comprehension of the intricate involvement of microglia in AD pathology by describing the crucial signaling pathways that dictate microglial responses to amyloid β (Aβ) and tau tangles. In particular, the authors discuss the significance of microglia-based risk factors, which hold promise for early detection and targeted therapeutic interventions during the pre-onset stages of AD.

Another review by Busch et al. in this SI discusses the impact of noncanonical isoforms of Aβ on major types of brain cells [14]. According to the amyloid hypothesis of AD pathology, Aβ is still considered a principal driver of the disease. Among the various oligomeric isoforms of Aβ, much of AD research conducted to date has been focused on Aβ_1–42_ and Aβ_1–40_. With meticulous attention to detail, the authors have expounded upon the synthetic pathways for the numerous isoforms of Aβ and described the intricate processes through which brain cells synthesize and process these manifold Aβ isoforms. The authors have also highlighted the potential of understudied isoforms of Aβ and provided substantial evidence for their impact on potentiating the ongoing neuroinflammatory process resulting in increased neurotoxicity.

The content of this SI is tailored to appeal to a wide audience encompassing both the basic and clinical neuroscience communities, fostering an inclusive exchange of knowledge and ideas. It provides invaluable insights into the pivotal involvement of glial cells in the intricate network of neuroinflammatory and neuroimmunological responses. It also sheds light on the recent and promising glia-based therapeutic strategies for treating neuroimmune disorders affecting the CNS.
